# Books and Authors

**Published:** 1907-10

**Authors:** 


### BOOKS AND AUTHORS.

**Modern Medicine.** Its Theory and Practice. In Original Contributions by American and Foreign Authors. Edited by **William Osler,** M.D., Regius Professor of Medicine in Oxford University, England; formerly Professor of Medicine in Johns Hopkins University, Baltimore. Assisted by Thomas McCrea, M.D., Associate Professor of Medicine and Clinical Therapeutics in Johns Hopkins University, Baltimore. In seven octavo volumes of about 1,000 pages each; illustrated. Volume I. Lea Brothers & Co., Philadelphia and New York. (Price per volume, cloth, $6.00; leather, $7.00; half morocco, $7.50, net prices).

These are the days of encyclopedic medicine; or, at least, of encyclopedias in medicine. Given a more or less distinguished editor and a group of more or less gifted writers, together with a courageous publisher and, presto, the deed is done. Following the lead of several eminent editors, Osler has entered the field to see what he can do to add to his already great fame as a teacher, author, and medical publicist. He has gone to England to live but maintains relations with America through frequent visits to his old home, but especially through his writings.

The list of contributors to the first volume of modern medicine numbers twenty-two including the editor, most of whom are known to the medical world. We add the names and topics, which will give the intending purchaser a good idea of the scope of the work.

Introduction—History and forecast of medicine, by William Osler, M.D., regius professor of medicine in Oxford University, England.

Part I—Predisposition and Immunity. Chapter I—Inheritance and disease, by J. George Adami, M.D., F.R.S., professor of pathology in McGill University, Montreal.

Part II—Diseases caused by physical agents. Chapter II—Light, *x*-rays, electricity; Chapter III—Air; Chapter IV—Heat and Cold, by Alfred Gordon, M.D., associate in mental and nervous diseases in the Jefferson Medical College, Philadelphia.

Part III—Diseases caused by chemical agents. Chapter V—Chronic lead poisoning; Chapter VI—Chronic arsenic poisoning; Chapter VII—Other metallic poisons, etc.; Chapter VIII—Poisoning from carbon monoxide, illuminating-gas, combustion products, carbon bisulphide, by David L. Edsall, M.D., assistant professor of medicine, University of Pennsylvania, Medical Department, Philadelphia.

Part IV—Diseases caused by organic agents. Chapter IX—Alcohol; Chapter X—Opium, morphinism, cocaine, by Alexander Lambert, M.D., professor of clinical medicine, Cornell University Medical College, New York. Chapter XI—Foods. Milk, fish, meat, etc., by Frederick G. Novy, M.D., professor of bacteriology in the University of Michigan, Ann Arbor. Chapter XII—Snake venoms, by Dr. Hideyo Noguchi, assistant at the Rockefeller Institute for Medical Research, New York. Chapter XIII—Auto-intoxications; Chapter XIV—Intoxications—protein, purin, carbohydrate and fat metabolism, by Alonzo Engelbert Taylor, M.D., professor of pathology, University of California, Medical Department, San Francisco.

Part V—Diseases caused by vegetable parasites. Chapter XV—Actinomyces; Chapter XVI—Aspergillus, by James Homer Wright, M.D., assistant professor of pathology in the medical school of Harvard University, Boston.

Part VI—Diseases caused by protozoa. Chapter XVII—Protozoa, by Gary N. Calkins, Ph.D., professor of protozoölogy in the Columbia University, New York City. Chapter XVIII—Mosquitoes, by L. O. Howard, Ph.D., chief, bureau of entomology of the United States Department of Agriculture. Chapter XIX—The malarial fevers, by Charles F. Craig, M.D., first lieutenant and assistant surgeon in the United States Army. Chapter XX—Black-water fever, by J. W. W. Stephens, M.D., Walter Myers lecturer on tropical medicine in the University of Liverpool, England. Chapter XXI—Trypanosomiasis, by David Bruce, C.B., F.R.S., D.Sc., M.B., C.M., (Edin.), Colonel, British Army. Chapter XXII—Amebic dysentery, by Richard P. Strong, M.D., director of the biological laboratory, Manila, P. I.

Part VII—Diseases caused by animal parasites. Chapter XXIII—General discussion; Chapter XXIV—Tetramode or fluke infection; Chapter XXV—Tæniasis—Cestode infection; Chapter XXVI—Round-worm infection—Nemathel-minthes; Chapter XXVII—Leeches, acariasis, tongue worm infections, myriapoda, etc., by Charles Wardell Stiles, Ph.D., D.Sc., chief of the division of zoölogy in the hygienic laboratory, United States Public Health and Marine Hospital Service, Washington, D. C.

Part VIII—Nutrition. Chapter XXVIII—General considerations of metabolism, normal and in disease, by Russell H. Chittenden, Ph.D., LL.D., professor of physiological chemistry in the Sheffield Scientific School of Yale University, New Haven, Conn., and Lafayette B. Mendel, Ph.D., professor of physiological chemistry in the Sheffield Scientific School, Yale University.

Part IX—Constitutional diseases. Chapter XXIX—Diabetes mellitus; Chapter XXX—Diabetes insipidus; Chapter XXXI—Gout, by Thomas B. Futcher, M.B., associate professor of medicine, Johns Hopkins University, Baltimore. Chapter XXXII—Obesity, by James M. Anders, M.D., professor of theory and practice of medicine and clinical medicine in the medico-chirurgical college, Philadelphia. Chapter XXXIII—Rickets, by George Frederic Still, M.A., M.D., (Cantab.), F.R.C.P. (Lond.), professor of diseases of children in King's College, London, Eng.

Chapter XXXIV—Scurvy, by Robert Hutchison, M.D., F.R.C.P. (Lond.), assistant physician to the London Hospital and to the hospital for sick children, Great Ormond street, London, Eng.

**A Treatise on the Principles and Practice of Medicine.** By **Arthur R. Edwards,** M.D., Professor of the Principles and Practice of Medicine and Clinical Medicine in the Northwestern University Medical School, Chicago. Octavo, 1328 pages, with 101 engravings and 19 plates. Lea Brothers & Co., Philadelphia and New York, 1907. (Cloth, $5.50, leather, $6.50, net prices.)

It would not appear to the careful observer that another work on practice were needed at the present time; nevertheless, each treatise possesses special merits of its own and to obtain a perfect whole the several parts must be collected and systematically arranged. One author writes from a view of one side of the shield, while another looks on the reverse to obtain his inspiration.

The work before us is a well-arranged, compact, systematic treatise on medicine prepared by an experienced teacher and will command, beyond doubt, the respectful attention of every physician who takes interest in internal medicine. Edwards has taught medicine for many years in the medical school of Northwestern University, Chicago, and has displayed his ability as a teacher by commanding a constantly increasing student clientele.

He, therefore, has written, as might be expected, an essentially clinical treatise, dealing with causative pathology, giving reasons for facts rather than painting typical pictures of disease. Treatment receives more attention than common, which is well, for, after all, the end purpose of the physician is the successful treatment of disease.

The author has paid due attention to the physiological action of drugs,—a very important topic that lays the foundation for the entire therapeutic superstructure. It is the whole purpose of this work to teach the student and practitioner how to treat disease scientifically with reference to cure if possible, or alleviation if this may only be. The twelve sections of the book consider in their order specific infections, diseases of the circulation, diseases of the respiratory tract, diseases of the digestive tract, diseases of the kidney, diseases of the blood, diseases of the ductless glands, constitutional diseases, diseases of the nervous system, intoxications, sunstroke, and diseases due to animal parasites. This is an admirable arrangement and enables both author and reader to grasp the inception of disease from the primitive invasion, leading through the different processes in the several organs and tissues, to the final consideration of animals that subsist on the human body and cause certain diseases or irritations.

We are of the opinion that Edwards has produced a book, all things considered, worthy a permanent place in the lore of medicine.

---

**Manual of Diseases of the Nose, Throat, and Ear.** By **E. B. Gleason,** M.D., Clinical Professor of Otology in the Medico-Chirurgical College of Philadelphia. 12 mo. pp, 556. Illustrated. Philadelphia and London: W. B. Saunders Company. 1907. (Price, flexible leather, $2.50 net.)

These are days when every college professor of a specialty in medicine deems it his bounden duty to write a book, the outcome of which is the great danger of an overstocked market. The redeeming feature of this condition, however, is that each book has some special points of quality not possessed by any other, hence the practitioner, author, teacher, and sometimes the student finds it important if not necessary to possess nearly all the books on the specialties. Coming now to the work in immediate question, we must confess that Gleason has prepared an attractive manual that must claim the respectful attention of the specialist as well as the general practitioner. He pays attention to instrumentation with marked exactitude,—a very important feature of this specialty because as much of diagnosis as well as treatment depends on deftly handled instruments.

The methods of treatment advocated by the author are, in general, of the simpler variety, though we confess to some surprise that he advises throwing into the antral sinus peroxide of hydrogen even when diluted with equal parts of Dobell's solution. Experience teaches that milder forms of irrigation serve quite as well and cause far less pain. All the operative procedures advised by Gleason are sanctioned by authority as well as the experience of the most disciplined surgeons in this line of work. We must not omit to mention a useful group of formulas placed at the end of the volume, which will prove an aid in the treatment of these maladies. The engravings are clear, and for the greater part original, while the printer has done his work so well that nothing could add to the beauty of the volume. We commend it to student, practitioner and specialist.

---

**Physical Diagnosis.** With case examples of the Inductive Method. By **Howard S. Anders,** A.M., M.D., Professor of Physical Diagnosis in the Medico-Chirurgical College, Philadelphia. Octavo, pp. 475. With 88 illustrations in the text and 32 plates. New York and London: D. Appleton and Company. 1907. (Price, $3.00.)

It is next to impossible to overrate the importance of a knowledge of the art and science of physical diagnosis; it is a requisite part of the equipment of a physician, and forms the foundation of success in practice. Because of such exactitude at the present day in laboratory methods it will not answer to neglect clinical investigation; indeed, the latter becomes even more necessary because of the former. Anders, the younger, has exhibited a knowledge of his subject, as might be anticipated, and has brought out its essentials in bold relief such, for example, as inspection, mensuration, percussion, the use of the stethoscope, and auscultation. There is danger that percussion, so much to be relied upon formerly, will become a lost art in the presence of the dazzling opportunities of the laboratory; but Anders points out its importance and shows its methods of application.

The use of the *x*-ray in diagnosis is described and illustrated by numerous unusually good skiagraphs, which the author designates with the abominable term, Röntgenograms. By and large, however, the book is beyond captious criticism; the text illustrations are excellent; while not only in general but even in particular, it takes rank among the better manuals of physical diagnosis published since Da Costa entered this field half a century ago.

---

**Progressive Medicine,** Vol. 1, No. 2, June, 1907. A Quarterly Digest of Advances, Discoveries and Improvements in the Medical and Surgical Sciences. Edited by **Hobart Armory Hare,** M.D., Professor of Therapeutics and Materia Medica in the Jefferson Medical College of Philadelphia. Octavo, 381 pages, with illustrations. Lea Brothers & Co., Philadelphia and New York. (Per annum, in four cloth-bound volumes, $9.00; in paper binding, $6.00, carriage paid to any address).

This periodical is constantly growing in value, the present number being one of the best ever issued. The first article is on hernia and is written by William B. Coley, of New York; the second is on surgery of the abdomen, exclusive of hernia, by Edward Milton Foote, of New York; the third, on gynecology, is by John G. Clark, of Philadelphia; the fourth, on diseases of the blood, etc., is by Alfred Stengel, also of Philadelphia; and the fifth, on ophthalmology, is by Edward Jackson, of Denver.

Coley gives a description of Graser's operation for umbilical hernia which is important, this being one of the most difficult forms of hernia in which to obtain surgical cure. The article is excellently illustrated and is a valuable contribution to the literature of that form of hernia. Foote's digest of the surgery of the abdomen deserves mention for its completeness, and for the excellent quality of its illustrations. Another section of the book that attracts attention is Clark's excerpts on gynecology. It takes up many of the newer questions relating to this topic and will prove of instructive interest to gynecologists. Whoever fails to subscribe for "Progressive Medicine" misses one of the most complete and satisfactory digests of medical literature yet published.

---

**International Clinics.** A Quarterly of Illustrated Clinical Lectures and especially prepared articles on Treatment, Medicine, Surgery, Neurology, Pediatrics, Obstetrics, Gynecology, Orthopedics, Pathology, Dermatology, Ophthalmology, Otology, Rhinology, Laryngology, Hygiene and other topics of interest to students and practitioners. Edited by **W. T. Longcope,** M.D. Volume II, seventeenth series. Philadelphia and London: 1907. J. B. Lippincott Co. (Cloth, $2.00).

The contributors to this number are Bertram Abrahams, John A. Bodine, Charles W. Burr, H. S. Clogg, Rufus I. Cole, T. D. Crothers, Charles Greene Cumston, Jean Dardel, Joseph DeLee, W. E. Carnegie Dickson, Professor Dieulofoy, George Dock, Simon Flexner, Alfred Gordon, Smith Ely Jelliffe, Maurice Letulle, Cuthbert Lockyer, Francis H. A. Marshall, Chauncey D. Palmer, Godfrey R. Pisek, Thomas Morgan Rotch, Francis Peyton Rous, John Madison Taylor, Joseph Edgar Tyree, and John W. Wainwright.

Four articles are devoted to "Treatment;" four to "Medicine;" four to "Surgery;" four to "Gynecology;" two to "Pediatrics;" four to "Neurology;" and three to "Pathology." It is a splendid number, well illustrated, and full of helpful suggestions of treatment. Two or three papers, in particular, merit comment,—Dr. Wainwright's on "Asepsis and Antisepsis," and Dr. DeLee's on the treatment of post-partum hemorrhage. The latter is one of the most complete and scientific expositions of the subject published in recent years and is beautifully illustrated in colors. Dr. Cumston's paper on "Surgical syphilis," also claims attention as a scholarly setting forth of an important topic.

---

**The Johns Hopkins Hospital Reports.** Vol. XII. Studies in Urological Surgery. Vol. XIV. Studies on Hypertrophy and Cancer of the Prostate. Baltimore: The Johns Hopkins Press. 1906.

The thirteenth volume of these valuable reports is devoted to studies in urological surgery by Hugh H. Young, one of the most accomplished genitourinary surgeons of the period. It is a remarkable volume, well printed and contains many original illustrations.

The fourteenth volume, by the same author, comprises studies on hypertrophy and cancer of the prostate. It is uniform in printing and mechanical excellence with the other, and like it, is well illustrated. Every physician interested in these subjects who would grow apace with progress should obtain these books.

---

**Medical Epitome Series. Diseases of the Nose and Throat.** By **J. B. Ferguson,** M.D., Instructor in Diseases of the Nose and Throat in the N. Y. Post-Graduate Medical School. 12mo., 243 pages, with 114 engravings. Edited by **Victor C. Pedersen,** M.D., Lecturer in Surgery at the New York Polyclinic Medical School and Hospital. Philadelphia and New York: Lea Brothers & Co. (Price, $1.00 net).

This is a very instructive little book and can be read with interest, even instructively, by the advanced practitioner. By this we mean to convey the impression that it is more than a mere quiz compend, and is well worthy examination and study by the all around physician in active practice, as well as by the undergraduate for whom it is more particularly designed. It contains many illustrations of merit, and is a credit to both author and editor.

---

**Essentials of Chemistry and Toxicology for the Use of Students in Medicine.** By **R. A. Witthaus,** A.M., M.D., Professor of Chemistry, Physics and Toxicology in Cornell University. Thirteenth edition. Revised by R. J. E. Scott, author of "The State Board Examination Series." New York: William Wood and Company. 1907. (Price, $1.00.)

When it is remembered that this reminder of the essentials of chemistry has passed through thirteen editions, the demand for it will be appreciated. This edition has been rearranged in part and rewritten in other part, so in considerable portion it is practically a new book. It is, too, one of the best chemistry compends extant, fulfilling all the requirements of such a book. Professor Witthaus is a veteran teacher, an expert in his specialty, and a writer who wields a facile pen,—all of which contribute to excellence in bookmaking.

